# Carbon‐Supported NiZnO Nanoparticles for Electrochemical Reduction of CO_2_ to Hydrocarbons

**DOI:** 10.1002/cphc.202500486

**Published:** 2026-06-05

**Authors:** Matt L. J. Peerlings, Floris S. J. van Seters, Nienke L. Visser, Alexander P. Flick, Erik Betz‐Güttner, Ramon van Maanen, Emiel J. M. Hensen, Petra E. de Jongh, Peter Ngene

**Affiliations:** ^1^ Materials Chemistry and Catalysis, Debye Institute for Nanomaterials Science Utrecht University Eindhoven Netherlands; ^2^ Laboratory of Inorganic Materials and Catalysis, Department of Chemical Engineering and Chemistry Eindhoven University of Technology Eindhoven Netherlands

**Keywords:** bimetallic nickel‐zinc catalyst, carbon support, CO_2_ electroreduction, electrocatalysis, stability

## Abstract

A compelling approach to close the carbon cycle is electrochemical CO_2_ reduction. Recently, it was demonstrated that polarized Ni catalysts can form value‐added hydrocarbon products like ethylene in this reaction. However, the competing hydrogen evolution reaction significantly lowers their selectivity. We investigate the effects of ZnO on the catalytic performance of well‐defined carbon‐supported NiO nanoparticles of 5–6 nm. Adding small amounts of ZnO to the highly dispersed NiO nanoparticles (in 20:1 Ni:Zn ratio) initially improves the Faradaic efficiency to C_1−3_ hydrocarbons from 4.6% to 5.7% at −1.1 V vs RHE, but adding more ZnO (10:1 Ni:Zn) favors the competing hydrogen evolution reaction. CO was detected only for the ZnO catalyst, suggesting electronic interaction between the nickel and zinc (oxide). After 2 h, the NiO catalyst outperforms the NiZnO catalysts in hydrocarbon selectivity. We attribute this to ZnO_x_ promoting the hydrocarbon selectivity, whereas full ZnO reduction over time results in the formation of a NiZn alloy that favors hydrogen formation. Structural characterization after catalytic testing shows leaching of zinc but a high structural stability for the nickel nanoparticles. These results demonstrate that electronic promotion offers an interesting approach to modulate the catalytic performance of nickel toward long‐chain hydrocarbons.

## Graphical abstract

Electronic interaction between nickel and zinc (oxide) in highly dispersed NiO‐ZnO nanoparticles on carbon support leads to improved Faradaic efficiency to C_1−3_ hydrocarbons in CO_2_ electroreduction at low ZnO concentrations (20:1 Ni: Zn ratio). Higher ZnO content (10:1 Ni:Zn ratio) favors the competing hydrogen evolution reaction, while only CO was detected for the ZnO‐only catalyst.

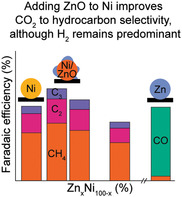

## 1 Introduction

The widespread use of fossil resources leads to excessive anthropogenic CO_2_ emissions, which cause climate change. To avert the use of fossil resources and mitigate these emissions, technologies that allow for CO_2_ to be captured and utilized are required to close the carbon cycle. Multiple strategies are possible, of which the electrochemical CO_2_ reduction reaction (CO_2_RR) offers the promise of converting CO_2_ and H_2_O to value‐added compounds while requiring mild reaction conditions and using renewable electricity [1]. Different products can be formed in this reaction, depending on the metal catalyst used. For example, CO is favored on Ag, Au, and Zn, whereas HCOOH is formed on Sn, Bi, and Pb. Other metals like Ni, Fe, and Pt typically produce mainly H_2_ in the competing hydrogen evolution reaction (HER) [2, 3]. Traditionally, Cu stands out as the only metal that performs C–C coupling and produces value‐added C_2+_ products like ethylene and ethanol in significant amounts [4].

Improving the catalytic performance of Cu‐based electrodes to C_2+_ products has been the focus of many CO_2_RR studies in the literature. This has led to important insights, such as *CO being a critical intermediate in the reaction [5]. Additionally, many strategies have been developed to improve the C_2+_ product selectivity by increasing the surface roughness [6], using oxide‐derived Cu [7], or increasing the *CO coverage via spillover by the addition of a CO‐producing metal [8, 9, 10, 11, 12]. Despite these efforts, copper still faces inherent issues associated with high overpotentials and low structural stability during CO_2_RR [13, 14]. These challenges must be addressed before industrial application is feasible.

While most research efforts are geared toward overcoming the aforementioned limitations of Cu‐based electrocatalysts, a different strategy is to explore alternative CO_2_RR electrocatalysts for the production of C_2+_ compounds. Some recent studies have shown that such Cu‐free alternatives exist, including, for example, nickel‐supported molecular iron tetraphenylporphyrin [15], imidazolium‐functionalized Mo_3_P [16] and polarized cobalt [17], and nickel catalysts [18]. However, in contrast to the vast literature on Cu‐based catalysts, the applicability of Cu‐free catalysts for C_2+_ production is still underexplored.

Zhou et al. [18] recently demonstrated that polarized Ni catalysts exhibit a different reactivity than Cu catalysts, resulting in the formation of up to C_6_ hydrocarbon products. However, the selectivity was low due to the competing HER being dominant on the Ni surface. They proposed a reaction mechanism analogous to Fischer‐Tropsch synthesis: *COOH + *CH_x_ coupling, followed by successive *CH_x_ insertions [19]. Crucial for this reactivity is the presence of polarized Ni^δ+^ species, because they suffer less from CO poisoning than metallic nickel and hence enable the formation of further‐reduced CO_2_RR products. By starting with different nickel salts to stabilize Ni^δ+^ species during CO_2_RR, they achieved a C_2+_ hydrocarbon selectivity above 10% at −1.2 V using phosphate‐derived nickel catalysts [18].

Preikschas et al. [20] showed that methane formation is favored at higher overpotentials and alkaline pH on phosphate‐derived Ni, whereas longer‐chain hydrocarbons are favored at lower overpotentials and near‐neutral pH. In addition, they showed that oxygenated products are also formed on these catalysts, including, among others, formate, methanol, ethanol, and *n*‐propanol. Recent work by Vos et al. [21] showed that the rate‐determining step for C_2+_ products is likely a hydrogenation step. As a result, a proton‐donating electrolyte (phosphate instead of bicarbonate) increases the reaction rate. These works demonstrate that Ni‐based catalysts offer an interesting alternative for producing C_2+_ compounds. However, they suffer from low CO_2_RR selectivity to these C_2+_ compounds because of the competing HER. Hence, strategies to improve the selectivity toward C_2+_ products should be explored.

Theoretical work has shown that combining high *CO binding energy metals like Ni with low *CO binding energy p‐block elements like Ga, Al, P, or Zn can yield CO_2_RR catalysts that form multicarbon products [22, 23]. It is important to note that this concerns intermetallic catalysts, in which both elements are intimately mixed and in a fully metallic state. This is different from the previously discussed work of Zhou et al. [18], where the *CO binding energy was decreased by stabilizing polarized Ni species. These theoretical studies have been verified by experimental work, showing that indeed nickel‐gallium (NiGa, Ni_3_Ga and Ni_5_Ga_3_) [24], nickel‐aluminum (Ni_3_Al) [25, 26] and nickel phosphide [27] are capable of producing C_2+_ products. Inspired by these findings, Van den Berg et al. [28] attempted to generalize these results by screening six intermetallic alloys based on a combination of strong and weak CO‐binding metals. They found that AlFe, AlNi, FeGa_3_, FeZn_4_, and NiGa all produced hydrocarbons like methane and ethylene, but with low selectivity due to the competing HER being dominant [28]. Another interesting intermetallic alloy is NiZn [29, 30, 31], where Zn is soluble in the Ni fcc structure up to about 21 atom% [32]. Besides Zn having a low *CO binding energy, recent work in our group [33] and by others [34] has shown that the presence of ZnO_x_ stabilizes cationic copper species during CO_2_RR, influencing their selectivity. Inspired by these results, we postulate that the addition of ZnO to nickel catalysts could stabilize polarized nickel species (due to the sluggish reduction of ZnO), possibly preventing CO poisoning or suppressing the HER.

In this work, we discuss the catalytic performance of NiO nanoparticles supported on a high‐surface‐area carbon and the effects of ZnO addition. It is well known that the particle size and shape of catalysts have a profound influence on their catalytic activity. Therefore, although carbon is known to produce hydrogen, carbon‐supported nanoparticles were chosen for this study because the preparation method gives rise to highly dispersed and well‐defined nanoparticles [35, 36]. Using this method, we successfully prepared highly dispersed and well‐defined 5 nm NiO and NiZnO nanoparticles as model catalysts to investigate the effect of atomic composition on the electrocatalytic CO_2_RR performance to hydrocarbon products, without the compromising effects of particle size or shape. Interestingly, adding zinc in a 20:1 Ni:Zn ratio improved the initial selectivity to hydrocarbon products, whereas a higher zinc content lowered the selectivity. Although zinc is prone to leaching, a high catalyst stability is demonstrated, which can be correlated to a high structural stability of the nickel nanoparticles. This work demonstrates that electronic modification of nickel catalysts is an interesting strategy to modify their catalytic performance.

## 2 Experimental

### 2.1 Chemicals

Graphite nanoplatelets (GNP‐500) were purchased from XG Sciences. Absolute ethanol and nitric acid (HNO_3_, 65%) were acquired from VWR. Nickel(II) nitrate hexahydrate (Ni(NO_3_)_2_·6H_2_O, ≥97.0%), zinc(II) nitrate hexahydrate (Zn(NO_3_)_2_·6H_2_O, ≥99.0%), anhydrous isopropanol (99.5%), potassium hydroxide (KOH, ≥85%), potassium bicarbonate (KHCO_3_, ≥99%), Chelex 100 sodium form (50–100 mesh), dimethyl sulfoxide (DMSO, ≥99.9%) and phenol (≥99.5%) were purchased from Sigma–Aldrich. Sustainion XA‐9 dispersion (5 wt%) was acquired from Dioxide Materials. Deuterium oxide (D_2_O, 98%) was bought from Buchem B.V. All aqueous solutions were prepared with Milli‐Q water (≥18.2 MΩ cm). All chemicals were used as received.

### 2.2 Synthesis of NiZnO‐GNP Catalysts

An incipient wetness impregnation procedure developed previously in our group [35] was used to prepare the NiZnO catalysts. Typically, 500 mg of GNP‐500 support was dried under dynamic vacuum for 120 min at 170°C. Aqueous impregnation solutions were prepared by dissolving 2.0 M nickel nitrate and 0.1, 0.2, and 0.4 M zinc nitrate in 0.1 M HNO_3_ to respectively yield 20:1, 10:1, and 5:1 Ni:Zn catalysts. Additionally, a ZnO/GNP reference catalyst was prepared from a 2.0 M zinc nitrate in 0.1 M HNO_3_ solution. The dried GNP‐500 support was impregnated with 0.79 mL g^−1^ of each metal precursor solution to achieve 90% pore filling (0.88 mL g^−1^ pore volume as determined using N_2_ physisorption). This was performed under static vacuum while stirring to ensure a homogeneous distribution over the support. The impregnated sample was then dried overnight under dynamic vacuum at room temperature.

The samples were heat‐treated to decompose the nitrate and obtain metal nanoparticles. First, 0.5 grams of sample was heated to 350°C for 90 min (2°C min^−1^) under 100 mL min^−1^ N_2_ flow. After cooling down to 40°C, the sample was reduced in 5% H_2_/N_2_ (100 mL min^−1^) by heating to 350°C for 90 min (2°C min^−1^) again. Once cooled down, the catalyst was slowly exposed to air to passivate the surface of the metal nanoparticles.

### 2.3 Preparation of Electrodes

Glassy carbon electrodes (SIGRADUR K discs, HTW Hochtemperatur‐Werkstoffe GmbH) were cleaned by overnight storage in 5% HNO_3_ solution and subsequent mechanical polishing using diamond polish suspensions with decreasing particle size of 1 µm, 0.25 µm, and 0.05 µm (MetaDi Supreme, Buehler). The polish residue was removed by sonication for at least 15 min in Milli‐Q water.

To fabricate the electrodes, first, a mixture of 5 mg catalyst, 1000 μL Milli‐Q water, 188 μL isopropanol, and 125 μL Sustainion dispersion was sonicated for at least 15 min. 350 µL of the as‐obtained catalyst ink was spray‐coated (Conrad HP‐200 Airbrush Pistol) onto each glassy carbon electrode to achieve a 0.2 mg/cm [2] loading. The fabricated electrodes were immersed for at least 24 h in 1.0 M KOH and subsequently rinsed with Milli‐Q to exchange the Cl^−^ for OH^−^ counterions before assessing the catalytic performance.

### 2.4 Structural Characterization

X‐ray diffraction (XRD) measurements on the catalyst powders before catalytic testing were performed on a Bruker D2 Phaser equipped with a Co K_α_ X‐ray source (*λ* = 1.79026 Å), whereas the electrodes after testing were measured on a Malvern Panalytical Empyrean equipped with a Mo K_α_ X‐ray source (*λ* = 0.71 Å). Scanning electron microscopy with energy‐dispersive X‐ray spectroscopy (SEM—EDX) images and maps were taken on a Zeiss EVO 15 operated at 20 kV and 500 pA, equipped with secondary electron detector. N_2_‐physisorption was measured at 77 K on a Tristar II Plus apparatus of Micromeritics after drying the sample at 170°C under vacuum for 14 h. The total pore volume was determined from the adsorption at *p* */p* *^0^* = 0.995.

High‐angle annular dark‐field scanning transmission electron microscopy (HAADF–STEM) was performed using a Thermo Fisher Talos F200X electron microscope operated at 200 kV to image the catalyst samples. EDX mapping was performed to assess the elemental distributions and quantify the atomic composition using the Ni and Zn K‐edges. Particle size diameters were determined by manually counting at least 200 nanoparticles using ImageJ analysis software. The number‐averaged particle diameter (*d*_n_) and Sauter mean diameter (*d*_s_) were calculated according to the following two formulas, respectively.

(1) $$d_{n}\pm \sigma_{dn}=\frac{1}{N}\sum_{i=1}^{N}d_{i}\pm \sqrt{\frac{1}{N}\sum_{i=1}^{N}{\left(d_{n}-d_{i}\right)}^{2}}$$

(2) $$d_{s}\pm \sigma_{ds}=\frac{\sum_{i=1}^{N}{d_{i}}^{3}}{\sum_{i=1}^{N}{d_{i}}^{2}}\pm \sqrt{\frac{1}{N}\sum_{i=1}^{N}{\left(d_{s}-d_{i}\right)}^{2}}$$

Thermogravimetric analysis (TGA) measurements were performed on a TA Instruments TGA‐5500 coupled to an MKS Cirrus 3 mass spectrometry (MS) system to assess the metal weight loading of the catalyst powders before testing. First, the samples were dried at 120°C (10°C/min, 30 min) under Ar flow (100 mL min^−1^). Next, the sample was cooled down to 30°C, and the gas flow was switched to 20% O_2_/Ar. The sample was then heated to 800°C (10°C/min, 10 min) to combust all carbon. After cooling down to 30°C, the metal weight loading was determined by comparing the mass before and after heating at the same temperature according to Equation (3):

(3) $$wt_{\text{NiZn}}=\left(m_{\mathrm{rel},\text{cat}}-m_{C}\right)\frac{xM_{\mathrm{Ni}}}{xM_{\mathrm{NiO}}+M_{\mathrm{ZnO}}}$$

With *m*_rel,cat_, and *m*_C_ indicating the relative mass (%) of the catalyst sample and pure GNP‐500 support after heat treatment, respectively. *x* represents the Ni:Zn molar ratio, and *M*_Ni_, *M*_NiO_, and *M*_ZnO_ are the average molar masses (g mol^−1^) of metal and metal oxide. All metal species are assumed to be oxidized in 1:1 stoichiometry with oxygen.

X‐ray photoelectron spectroscopy (XPS) measurements were performed using a Thermo Scientific K‐alpha spectrometer with a monochromatic Al‐Kα X‐ray source (Al Kα: hν = 1486.6 eV, power: 72 W). Powder samples were mounted on double‐sided carbon tape. Prior to spectral acquisition, the surface of the sample was etched with an argon‐ion beam (ion energy: 2000 eV, etch time: 30 s) to remove the layer of adventitious carbon. A flood gun was used during analysis to compensate for surface charging. Survey spectra were recorded with a pass energy of 200 eV and a step size of 0.5 eV, using 12 scans and a dwell time of 50 ms. XPS spectra were acquired with a pass energy of 50 eV and a step size of 0.1 eV, using 30 scans and a dwell time of 50 ms. Data analysis was performed using the CasaXPS software. The reference line for energy calibration was the C 1s line set at 284.8 eV. Background subtraction was conducted using a Shirley background, and peak fitting was carried out using a GL(30) line shape.

### 2.5 Electrocatalytic Experiments

A custom‐built H‐cell (Figure S1) was used for all electrochemical experiments. It was cleaned thoroughly between measurements by storing overnight in 5% HNO_3_ and rinsing with Milli‐Q. Both the cathode and anode compartments were filled with 15 mL of 0.1 M KHCO_3_ electrolyte, separated by a Fumasep FAA‐3‐PK‐130 anion exchange membrane (Fumatech BWT GmbH). Chelex (100 sodium form, Sigma–Aldrich) was added to the electrolyte prior to usage to remove any metal impurities. The cathode and anode compartments were flushed 30 min prior to and during electrolysis using 20 mL min^−1^ of CO_2_ (Linde, purity 5.3) and Ar, respectively. In addition, the cathode compartment was stirred at 400 rpm during electrolysis. A three‐electrode configuration featuring an Ag/AgCl reference electrode in 3 M KCl (Metrohm) and commercial IrO_2_‐based anode (Dioxide Materials) was used in combination with a Parstat potentiostat. The working and counter electrodes exposed 3.8 cm^2^ geometric surface area. All reported electrode potentials were converted to RHE by accounting for the catholyte pH of 6.8 and performing an iR‐correction according to the following formula:

(4) $$E_{\mathrm{iR}}\text{vs RHE}(V)=E_{\mathrm{Ag}/\text{AgCl}}+0.210+0.059*pH-iR$$

The Ohmic resistance was determined as the average of values obtained from electrochemical impedance spectra taken before and after electrolysis, while verifying that the resistance stayed constant throughout the experiment. The catalyst performance was assessed using chronoamperometry with 85% iR compensation applied on‐the‐fly, with the remainder corrected afterward.

An online gas chromatograph (Global Analysis Solutions Microcompact GC 4.0) with high‐purity nitrogen (N_2_; 99.999%) as carrier gas was used for gaseous product quantification using three channels. The first channel was equipped with an Rt‐QBond (10 m*0.32 mm, Agilent) packed column and a flame ionization detector (FID) for detection of C_1–3_ hydrocarbon molecules; the second channel with a Molecular Sieve 5 A (10 m* 0.53 mm, Restek) packed column and an FID detector coupled with a methanizer for CO detection; and the third channel with a Carboxen 1010 (8 m*0.32 mm, Agilent) packed column and TCD detector for detection of H_2_. Electrolyte samples were taken after each potential. Products in the liquid phase were quantified by measuring sample tubes filled with 500 µL electrolyte and 100 µL of an internal standard containing 10 mM DMSO and 50 mM phenol in D_2_O on a 400 MHz VNMRS‐400 Varian NMR.

### 2.6 Determination of Electrochemical Surface Area (ECSA)

Prior to catalytic testing at increasingly cathodic potentials, the electrochemical surface area (ECSA) was assessed by performing double‐layer capacitance (DLC) measurements between +0.6 and +1.3 V vs RHE at scan rates of 200–1400 mV/s. The capacitance values were determined from the slope of an allometric fit of the hysteresis difference between the anodic and cathodic scans at +0.9 V vs RHE versus scan rate.

## 3 Results and Discussion

### 3.1 Structural Characterization of NiZnO/GNP Electrodes

A series of five different catalysts with systematically varied Ni:Zn atomic ratios was prepared via incipient wetness impregnation of an aqueous metal nitrate solution in a high surface area carbon support composed of graphite nanoplatelets (GNP‐500). The GNP‐500 support has a pore volume of 0.88 mL g^−1^ and Brunauer‐Emmett‐Teller (BET) surface area of 570 m^2^ g^−1^, as determined by N_2_ physisorption. After incipient wetness impregnation, a heat treatment was carried out to convert the metal nitrates to oxides, followed by reduction in 5% H_2_/N_2_ atmosphere. For a fair comparison, the Ni weight loading (total amount of Ni) was kept constant at 10 wt% for all NiO‐based catalysts, whereas ZnO was introduced in various amounts by increasing the concentration of zinc nitrate in the impregnation solution. Both thermogravimetric analysis (TGA) and inductively coupled plasma (ICP) spectroscopy confirm that the Ni loading of all NiO‐based catalysts and the Zn loading of the ZnO catalyst are close to the intended 10 wt%, as summarized in Table 1. In addition, ICP analysis confirms that the Ni:Zn atomic ratio is close to the intended 20, 10, and 5 to 1 for all NiZnO catalysts.

**TABLE 1:** Overview of catalysts and their respective properties.

Catalyst	Ni loading (wt%)		Zn loading (wt%)		Ni:Zn (at%)			**d**_**s**_ **± σ**_**s**_ **(nm)**	Crystallite size (nm)	** Ni reduction degree (%)** e
	**TGA** a	**ICP** b	**TGA** a	**ICP** b	**ICP** b	**XPS** e	**HAADF–STEM‐EDX** c	**HAADF–STEM** c	**XRD** d	**XPS** e
NiO	10.8	9.9	—		n.a.	n.a.	n.a.	5.0 ± 1.0	5	13.1
Ni_20_Zn_1_O	9.5	9.4	0.5	0.5	21:1	26:1	18:1	5.7 ± 1.5	5	16.5
Ni_10_Zn_1_O	10.2	9.0	1.0	0.9	11:1	8:1	9:1	5.3 ± 1.2	6	17.3
Ni_5_Zn_1_O	9.0	8.3	1.7	1.6	6:1	5:1	5:1	6.0 ± 1.5	5	16.7
ZnO	—	—	10.0	9.7	n.a.	n.a.	n.a.	n.a.	7	—

a Determined by TGA analysis, assuming the intended atomic ratios of 20, 10, and 5 Ni to 1 Zn for the NiZnO catalysts.

b Determined by ICP analysis.

c Obtained using HAADF–STEM‐EDX.

d HAADF–STEM with EDX analysis and obtained from the XRD patterns in Figure 2 by applying the Scherrer equation to the NiO(111) peak at 52° and the ZnO(103) peak at 75°.

e Determined via quantitative XPS analysis. The reduction degree is defined as Ni^0^/(Ni^0^ + Ni^2+^) and reflects the spectral contribution of Ni^0^. Ni^2+^ contributions are from NiO and Ni(OH)_2_.

**FIGURE 1:**
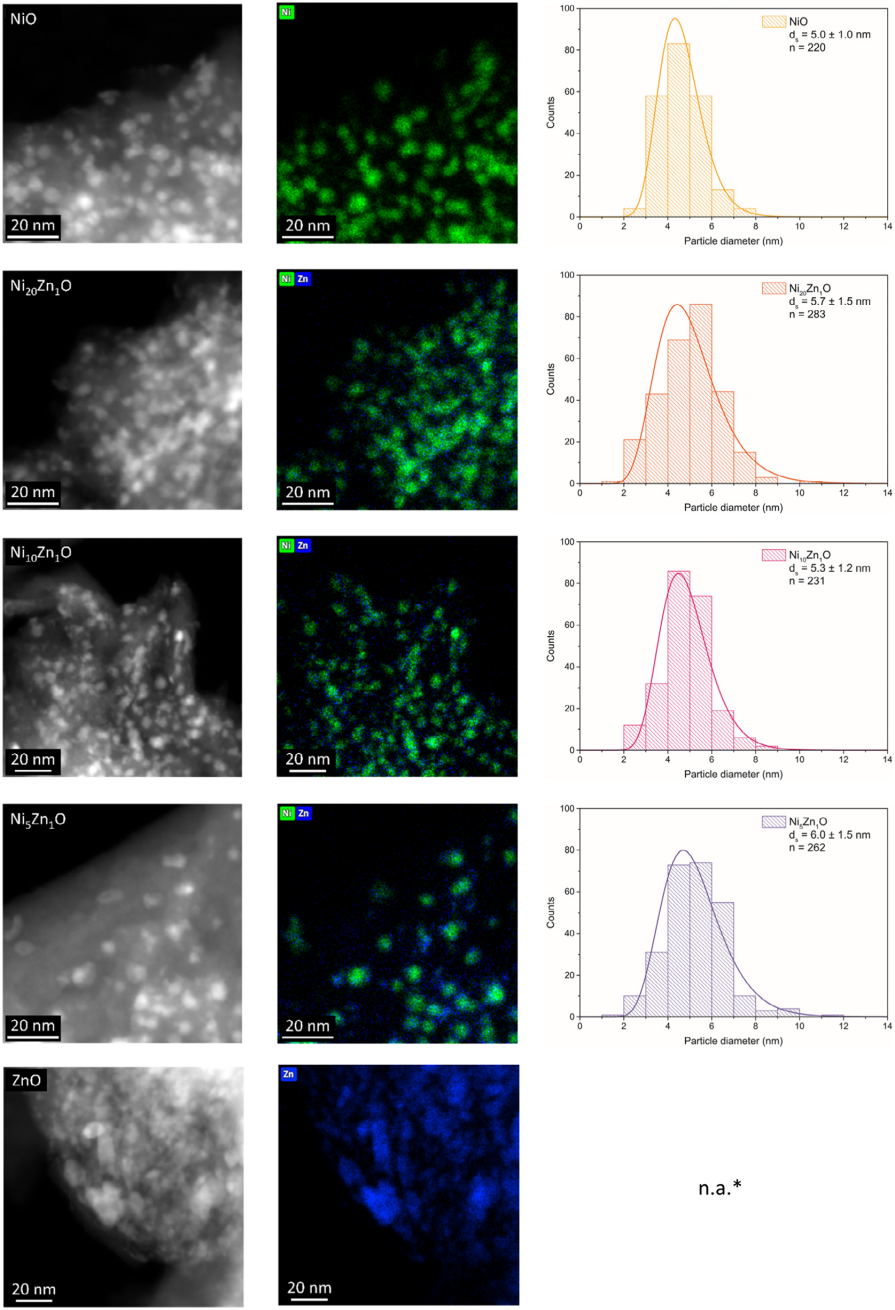
High‐resolution HAADF–STEM images (left) and EDX maps (middle), together with corresponding particle size distributions (right) of the different NiZnO samples. *The ZnO particles are not well‐defined and spherical, preventing accurate particle size determination.

Figure 1 shows representative high‐angle annular dark‐field scanning transmission electron microscopy (HAADF–STEM) images and corresponding Ni and Zn energy dispersive X‐ray spectroscopy (EDX) maps. By quantifying the EDX maps, the Ni:Zn ratio in Table 1 was obtained. These ratios agree well with the intended and the ICP values, again confirming the atomic composition of the catalysts. Analysis of the HAADF–STEM images yields the particle size distributions shown in Figure 1 and summarized in Table 1 and Table S1. These data show that the surface‐averaged particle sizes d_s_ range between 5 and 6 nm for all nickel‐containing catalysts. Therefore, we can conclude that the particle sizes are not affected by the addition of zinc.

The EDX maps show that the spherical particles on the support are composed of nickel. The zinc location is more difficult to assess due to the low signal associated with its lower weight loading. In the ZnO catalyst, the particles do not have a spherical shape and are less defined. This is likely due to incomplete reduction of ZnO to Zn during the thermal treatment in H_2_, as ZnO is difficult to reduce completely [37]. As a consequence, it was not possible to reliably determine the ZnO particle size distribution via HAADF–STEM analysis. However, qualitative analysis of the images shows that the particle sizes are in the same order of magnitude as those of the NiO particles.

**FIGURE 2:**
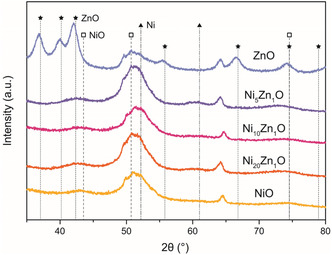
XRD patterns of the five NiZnO catalysts with different compositions, showing diffraction peaks corresponding to crystalline NiO and ZnO. The peaks at 65° correspond to the GNP‐500 support. The NiO(111) peak at 2*θ* = 43.5° and ZnO(103) peak at 2*θ* = 74.5° were used for crystallite size determination.

The XRD patterns in Figure 2 show diffraction peaks corresponding to NiO for all Ni‐containing samples. This is likely due to Ni passivation (oxidation) in air after the heat treatment in H_2_. Consequently, the obtained NiO nanoparticles likely consist of a Ni core surrounded by a NiO layer. Given that the diffraction patterns of all NiO‐containing catalysts are similar, we again conclude that the addition of ZnO does not impact the NiO particle size. This is confirmed by quantitative analysis of the NiO(111) peaks at 2*θ* = 43.5°, which yield NiO crystallite sizes of 5–6 nm, as summarized in Table 1. No ZnO‐related peaks are observed for the NiZnO catalysts, indicating that ZnO is either present as an amorphous or as a highly dispersed phase on the support. In contrast, for the Zn‐only catalyst, clear ZnO‐related peaks are observed. Quantitative analysis of the ZnO(103) peak at 2*θ* = 74.5° yields a ZnO crystallite size of 7 nm, which is in the same size range as the NiO particles.

To obtain more information on the reduction degree of nickel, XPS measurements were performed. Contributions from metallic Ni, NiO, and Ni(OH)_2_ were observed in all nickel‐containing catalysts, as shown in Figure S2. The surface Ni:Zn ratio and the Ni reduction degree were determined by fitting the XPS spectra (Table 1). The NiZnO catalysts contain more metallic Ni than the NiO sample. We speculate that ZnO protects metallic Ni from air exposure and reoxidation (Figure S3). This result strongly indicates that nickel and zinc are in close contact in the NiZnO catalysts. No impurities were detected in the XPS survey spectra (Figure S4).

Characterization confirms the successful synthesis of a series of NiZnO catalysts with controlled Ni:Zn ratios, all exhibiting uniform particle sizes in the range of 5–6 nm. The NiO catalyst contains a metallic Ni core, which is surrounded by a passivating NiO and Ni(OH)_2_ layer formed by exposure to air. Zinc is mostly present as amorphous and highly dispersed ZnO. In the NiZnO catalysts, nickel and zinc oxide are in close proximity, with ZnO forming a passivating layer that protects part of the nickel from oxidation upon exposure to air.

After synthesis of the five catalyst powders, a catalyst ink with Sustainion as a binder was spray‐coated onto glassy carbon electrodes. A representative SEM image of the NiO/GNP electrode in Figure S5 shows that the catalyst powder was deposited with a homogeneous distribution. Before catalytic testing, the electrochemically active surface area (ECSA) of the electrodes was determined by performing cyclic voltammetry (CV) measurements at increasing scan rates ranging from 40 to 1400 mV/s in a wide potential range of +0.6 to +1.3 V vs RHE. These anodic potentials were chosen to ensure that all metallic Ni was oxidized prior to catalysis, as oxide‐derived Ni is reported to be more selective to hydrocarbon products [18]. A representative voltammogram of the Ni_20_Zn_1_O/GNP electrode is shown in Figure S6. The hysteresis at +0.9 V vs RHE versus scan rate was used for ECSA estimation, yielding the DLC values in Figure S7 for all catalysts and a GNP‐500 reference. All obtained DLC values are similar, demonstrating that the capacitive response is dominated by the high surface area GNP‐500 support. Therefore, these values provide no information on the Ni ECSA. Instead, they indicate that the amount of catalyst powder deposited via spray coating is sufficiently reproducible for catalytic testing.

### 3.2 Catalytic Performance of Monometallic Ni, Zn, and GNP Electrodes

**FIGURE 3:**
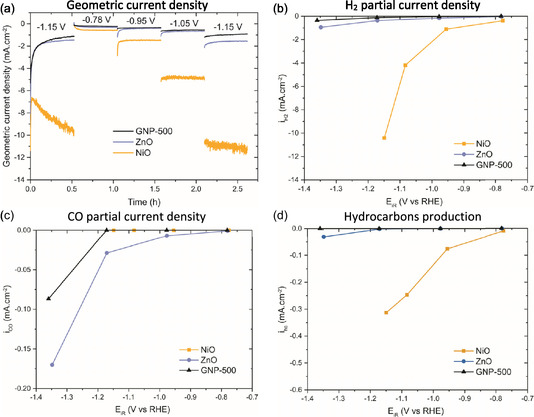
Electrocatalytic test results of the NiO, ZnO, and GNP‐500 electrodes. (a) Geometric current density as a function of time, recorded at five potentials (−0.75 to −1.3 V vs RHE). (b) Partial current density as a function of potential for H_2_; (c) CO; and (d) the sum of all detected hydrocarbon products (hc): methane, ethylene, ethane, propylene, and propane.

After preparation of the NiZnO‐based electrodes, their electrocatalytic performance was tested. First, the results of the NiO and ZnO electrodes will be compared to those of a reference electrode containing only GNP‐500 carbon and no metal nanoparticles. During catalytic testing, the electrodes were activated at −1.3 V vs RHE for 30 min and subsequently subjected to increasingly cathodic potentials from −0.75 V to −1.3 V vs RHE for half an hour at each potential. The respective chronoamperometry data are given in Figure 3a. At all potentials, the NiO electrode shows a much higher activity than the ZnO and GNP‐500 electrodes. This demonstrates that nickel is an active reduction catalyst. Please note that the iR‐compensated potentials of the different electrodes deviate slightly due to incomplete iR compensation (∼80%) during the chronoamperometry measurements, with the remainder corrected afterward. This is especially true for the ZnO and GNP‐500 electrodes, which are less active than the NiO electrode and hence are subjected to less iR compensation and more cathodic potentials.

**FIGURE 4:**
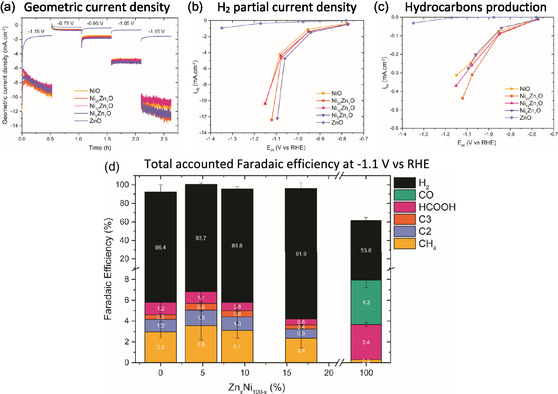
Electrocatalytic test results for the five NiZnO electrodes. (a) Geometric current density as a function of time, recorded at five potentials (−0.75 to −1.2 V vs RHE). (b) Partial current density as a function of potential for H_2_. (c) The sum of partial current densities for all detected hydrocarbon products (methane, ethylene, ethane, propylene, and propane) as a function of potential. (d) Faradaic efficiency of all accounted products as a function of atomic composition (expressed as Zn_*x*_Ni_100‐x_ content) at −1.1 V vs RHE.

**FIGURE 5:**
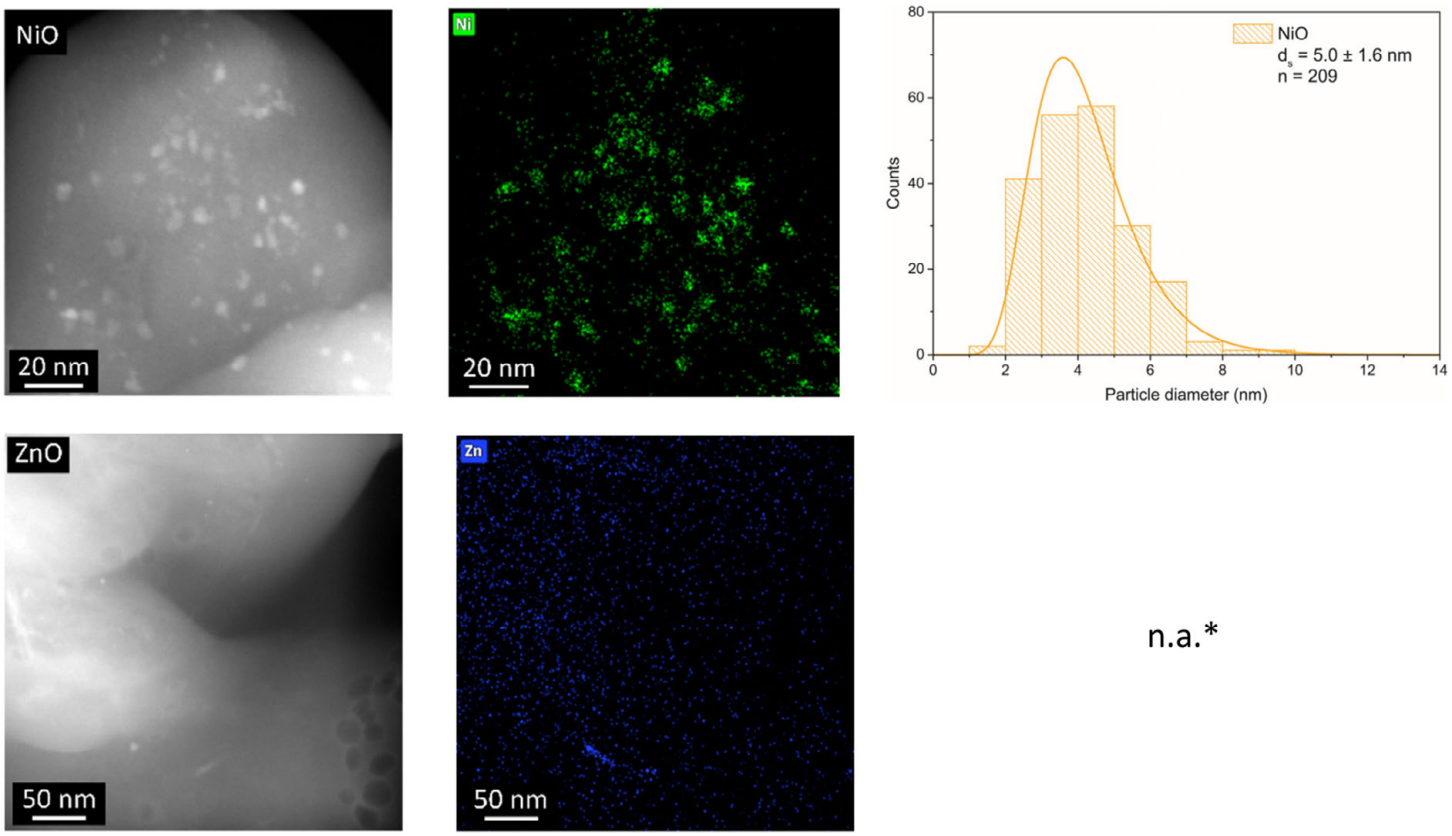
High‐resolution HAADF–STEM images (left) and corresponding EDX maps (middle), together with corresponding particle size distributions (right) of the NiO and ZnO catalysts after testing up to −1.2 V vs RHE. *The absence of well‐defined spherical particles for the ZnO sample made accurate particle size determination impossible.

The ZnO electrode shows a slightly higher activity (∼2x) than the GNP‐500 electrode for both H_2_ and CO production in Figure 3b and c, respectively. This indicates a low activity of ZnO_x_, likely because of incomplete ZnO reduction to the catalytically active Zn metal. Previous studies have reported that typically, ZnO reduction requires high overpotentials and long electrolysis time, depending on the morphology of the ZnO [33, 38, 39]. Incomplete ZnO reduction is corroborated by a low total accounted Faradaic efficiency (FE) of about 60% for the ZnO catalyst (Figure 4d). Additionally, leaching of Zn^2+^ into the electrolyte might also contribute to the low activity (Figure 5). These effects will be discussed in more detail in the next sections.

Figure 3b shows that the high activity of nickel is mainly due to the production of undesired H_2_, as expected for Ni. Interestingly, however, Figure 3d shows that the NiO electrode also produces hydrocarbons (hc). The detected hydrocarbon products include methane, ethylene, ethane, propylene, and propane, whereas longer‐chain hydrocarbons were below the detection limit of the gas chromatograph (GC). Although these current densities are smaller than those of H_2_, the production of these compounds encourages studying this Ni model system in more detail.

### 3.3 Catalytic Performance of NiZnO Electrodes

To investigate how the catalytic performance of Ni is influenced by ZnO_x_ addition, three NiZnO catalysts containing 20:1, 10:1, and 5:1 Ni:Zn atomic ratios were tested. The catalytic performance data are provided in Figure 4. Additionally, the partial current densities to CO and the hydrocarbon products per amount of carbon atoms (methane, C_2_, and C_3_) are included in Figure S8, whereas total accounted Faradaic efficiency values of the catalysts are given in Figures S9–11.

Figure 4a shows that all NiO‐containing catalysts exhibit a similar total current density. This reveals that adding ZnO does not affect the activity much, which is likely related to the previously discussed low activity of ZnO_x_ in this potential window. Interestingly, despite the similar total activity for the NiO‐containing electrodes, they differ significantly in terms of selectivity. The undesired HER is dominant for all NiO‐based catalysts, as the partial current density to H_2_ in Figure 4b is much larger than that to hydrocarbon products in Figure 4c. As a consequence, the H_2_ FE exceeds 80% for all NiO‐containing catalysts, as given in Figure 4d at −1.1 V vs RHE.

The H_2_ partial current density is highest for the Ni_5_Zn_1_O catalyst and lowest for the NiO and Ni_10_Zn_1_O catalysts. No clear relation between H_2_ partial current density and atomic composition is observed at −1.1 V vs RHE (Figure S12a). The Ni_20_Zn_1_O catalyst exhibits the highest partial current density to hydrocarbon products, whereas the Ni_5_Zn_1_O catalyst exhibits the lowest. The partial current density to hydrocarbon products shows an optimum atomic composition at −1.1 V vs RHE in Figure S12b: adding ZnO to NiO in a 20:1 atomic ratio improves the hydrocarbon production, whereas adding more ZnO leads to a decrease in selectivity. This causes a similar optimum in the hydrocarbon FE as function of atomic composition visible in Figure 4d. This optimum is striking because ZnO_*x*_ does not produce hydrocarbons nor influence the particle size of NiO. This implies that zinc interacts with nickel in such a way that the selectivity to hydrocarbon products is improved.

To better understand the interactions between nickel and zinc (oxide), it is important to note that no CO is observed for any of the NiZnO catalysts. This is surprising, as CO is the main CO_2_RR product for ZnO_x_ catalysts and single‐atomic Ni catalysts [39, 40, 41]. A possible explanation is the low activity of ZnO_x_ in combination with its low loading, being at least five times lower for the NiZnO catalysts than for the ZnO catalyst. However, assuming an equal CO activity and accounting for the difference in loading, we would still expect a CO partial current density of around 5 µA cm^−2^ for the Ni_5_Zn_1_O catalyst at −1.1 V vs RHE, which is above the detection limit of the GC. Another explanation for the absence of CO is that Ni and ZnO_x_ interact in such a way that the formation of CO is suppressed on the latter. This is in line with the observation that ZnO_x_ influences the selectivity of Ni to hydrocarbon products.

Whereas these results establish that Ni and ZnO_x_ interact, the nature of this interaction remains an open question. One possibility is CO spillover from ZnO_x_ to Ni sites, at which CO is further reduced to hydrocarbon products. Such a spillover mechanism is well‐known for CuZn catalysts [11, 12]. Although it is known that CO can be reduced to hydrocarbons on Ni‐based electrocatalysts, CO spillover has not been reported before [21]. However, CO spillover does not explain why adding more ZnO decreases the hydrocarbon selectivity.

Another possibility is that Ni and ZnO_x_ exhibit an electronic interaction that affects their catalytic performance. For instance, full reduction of Ni and Zn could lead to the formation of an intermetallic alloy phase [22, 23]. However, this is unlikely because of the sluggish reduction of ZnO [33, 38, 39]. Instead, we hypothesize that the intimate contact between Ni and ZnO_x_ retards the full reduction of NiO to Ni due to the slow reduction of ZnO_x_, which is in line with findings for CuZnO catalysts [33, 34]. As a result, polarized Ni^δ+^ species are stabilized, which has been proposed to prevent CO poisoning and promote hydrocarbon formation [18]. Analogously, H_2_ temperature‐programmed reduction (TPR) experiments have reported that the reduction peak of NiO shifts to higher temperature (NiO reduction becomes more difficult) due to the interaction between Ni and ZnO_x_ [42].

### 3.4 Structural Stability of NiZnO/GNP Electrodes

To obtain more information on the structural stability of the NiZnO catalysts, the electrodes were characterized after the catalytic tests. Interestingly, the SEM images of the electrodes in Figure S13 show no detachment of the carbon support nor large nickel or zinc agglomerates. Also, the XRD patterns in Figure S14 show no Ni‐ or Zn‐related diffraction peaks, confirming the absence of large crystallites. To visualize the NiZnO particles, HAADF–STEM was employed. The images and particle size distributions are shown in Figure 5 for the NiO and ZnO catalysts and in Figure S15 for the NiZnO catalysts. In addition, the particle sizes and Ni:Zn ratios are summarized in Table 2.

**TABLE 2:** Overview of the catalyst properties after testing as determined by HAADF–STEM and corresponding EDX analysis.

Catalyst	**d**_**n**_ **± σ**_**n**_ **(nm)**	**d**_**s**_ **± σ**_**s**_ **(nm)**	Ni:Zn
NiO	4.2 ± 1.3	5.0 ± 1.6	n.a.
Ni_20_Zn_1_O	4.1 ± 1.2	4.7 ± 1.4	53 : 1
Ni_10_Zn_1_O	4.5 ± 1.5	5.4 ± 1.7	50 : 1
Ni_5_Zn_1_O	4.1 ± 1.3	4.9 ± 1.6	17 : 1
ZnO	n.a.	n.a.	n.a.

No significant differences are observed in the particle size of the NiO‐containing catalysts before and after the catalytic tests, with the differences in both the number‐averaged and surface‐averaged particle size falling within the error margin. Therefore, we can conclude that the nickel particles exhibit high structural stability during CO_2_RR. This is quite different for the ZnO catalyst, for which significantly fewer particles are present after catalytic testing than before. This indicates a loss of zinc. A lower zinc content is also observed for the NiZnO electrodes. Whereas the Ni:Zn ratios before catalytic testing were close to 20, 10, and 5, after testing they increased to 53, 50, and 17, respectively. Care should be taken in the exact interpretation of these latter ratios due to the low EDX signal of Zn, causing a relatively high uncertainty in its quantification. Nonetheless, these results clearly show a loss of zinc during catalysis that contrasts with the high structural stability of nickel.

To verify the loss of zinc, the concentrations of nickel and zinc in the catholyte after the catalytic tests were determined using ICP. The results are summarized in Table S2. Both metals are detected, indicating that some catalyst detachment and/or metal dissolution occurred for all electrodes.

### 3.5 Electrocatalytic Stability of NiZnO/GNP Electrodes

**FIGURE 6:**
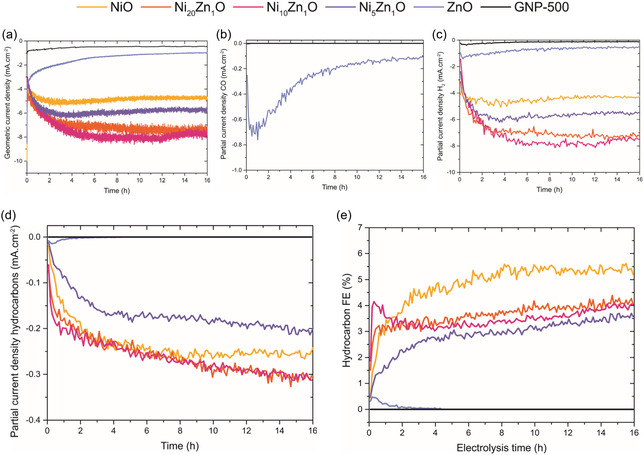
Electrocatalyst stability results of the NiZnO electrodes at −1.1 V vs RHE. (a) Total geometric current density, (b) partial current density to CO, (c) H_2_, and (d) hydrocarbon products and (e) Faradaic efficiency to hydrocarbon products as a function of time. Hydrocarbon products include methane, ethylene, ethane, propylene, and propane.

To obtain more information about the stability of the catalysts, the electrodes were subjected to 1.1 V vs RHE for 16 h. This potential corresponds to the previously observed potential with optimum hydrocarbon selectivity. Figure 6a shows the geometric current density of the five NiZnO electrodes and the GNP‐500 reference as a function of time. Starting with the ZnO electrode, it is interesting to see that the geometric current density keeps decreasing throughout the measurement, ultimately reaching only 1 mA cm^−2^ after 16 h of electrolysis. Despite this continuous decrease in total current, the production of CO increases in the first 2 h of electrolysis, after which it decreases, as shown in Figure 6b. The increase in CO production and CO FE to around 30% (Figure S16) in the first 2 h of catalysis is likely due to ZnO reduction, in line with the sluggish reduction of ZnO observed in other works [33, 38]. After 2 h of catalysis, the CO production decays, likely due to Zn dissolution. Indeed, the HAADF–STEM images of the ZnO electrode after 16 h of electrolysis in Figure S17 show almost no detectable quantities of zinc. Nevertheless, not all zinc is lost, as the activity to CO of the ZnO electrode remains higher than that of the GNP‐500 reference even after 16 h of catalysis.

The NiO‐containing electrodes exhibit more stable geometric current densities (Figure 6a). Activation is observed in the first 4 h of electrolysis, after which the geometric current densities are relatively stable throughout the remaining 12 h. The H_2_ partial current densities of the NiO‐based electrodes in Figure 6c follow a similar trend, with activation in the first 4 h followed by a stable catalytic activity. This is logical, given that H_2_ is the dominant product with an FE higher than 90% on all NiO‐based electrodes. Interestingly, activation is also observed for the partial current densities to hydrocarbon products in Figure 6d. For all NiO‐based electrodes, they improve throughout the 16 h of electrolysis, although the steepest increase is within the first 4 h. This interesting observation suggests that the NiO‐based catalysts activate rather than deactivate during CO_2_RR. Consequently, the hydrocarbon FE of all NiO‐containing catalysts (Figure 6e) also improves over time. Differences exist between the different NiZnO electrodes, with the NiO and Ni_5_Zn_1_O catalysts showing the largest increase in hydrocarbon FE in the first 4 h of electrolysis.

The remarkable activation of the NiO‐based catalysts during CO_2_RR might in part be explained by reduction of NiO to Ni. Catalyst restructuring (sintering or agglomeration) also does not seem to play a role, as the HAADF–STEM images of the tested electrodes (Figure S17) show no significant change in the catalyst particle size before and after the 16 h of electrolysis. In addition, the increasing hydrocarbon production might be related to changes in the coverage of reaction intermediates on the catalyst surface. Interestingly, the presence and concentration of ZnO_x_ influence the rate of this activation. Whereas the Ni_20_Zn_1_O and Ni_10_Zn_1_O electrodes start with a higher hydrocarbon FE than the NiO electrode, this changes after about 2 h. As a result, the NiO electrode unexpectedly shows the highest hydrocarbon FE during the prolonged electrolysis. Interestingly, the optimum CO activity of the ZnO electrode in Figure 6b is also around 2 h of electrolysis, at which time the ZnO is expected to be fully reduced to Zn. This suggests that the reduction of ZnO to the CO‐producing Zn does not benefit the selectivity of the NiZnO‐based catalysts to hydrocarbon products. Likely, ZnO reduction to Zn facilitates the formation of a NiZn alloy phase. Therefore, we deduce that this NiZn alloy phase promotes the competing HER, in accord with the higher H_2_ production for all NiZnO electrodes than for the NiO electrode.

The initial higher selectivity of the NiZnO catalysts might be explained by the presence of a Ni/ZnO_x_ interface. The fact that this interface disappears when ZnO is fully reduced to Zn explains why the hydrocarbon FE of the Ni_20_Zn_1_O and Ni_10_Zn_1_O catalysts are only higher than that of the NiO catalyst in the first 2 h of electrolysis. The higher FE of the NiO sample during prolonged CO_2_RR reveals for the first time that NiO leads long activation period to form the most hydrocarbon‐selective phase during CO_2_RR. Although more detailed in situ/operando studies using X‐ray absorption spectroscopy and Raman spectroscopy are required to unravel the catalytically active phase, the results presented here clearly show that the addition of ZnO_x_ can improve the hydrocarbon selectivity of carbon‐supported Ni nanoparticles during CO_2_RR. In addition, these results demonstrate that following the evolution of the catalyst performance during prolonged electrolysis can yield important insights into the catalyst's active phase.

## 4 Conclusions

We have studied the effect of ZnO addition on the performance of NiO electrocatalysts for CO_2_ reduction to hydrocarbons. Well‐defined 5 nm NiO nanoparticles supported on a high surface area graphitic carbon were prepared via incipient wetness impregnation. Adding ZnO to the NiO catalysts did not alter the particle size, allowing us to reliably study atomic composition effects. Remarkably, the addition of ZnO in a 20:1 Ni:Zn ratio improved the Faradaic efficiency (FE) of C_1–3_ hydrocarbon products from 4.6% for NiO to 5.7% for Ni_20_Zn_1_O at −1.1 V vs RHE, while higher zinc contents lowered the hydrocarbon FE. Interestingly, CO was only observed for the ZnO catalyst and not for the NiZnO ones, suggesting electronic interaction between Ni and ZnO_x_ that modifies their selectivity in electrochemical CO_2_RR. Remarkably, long‐term (16 h) catalytic tests revealed that the promoting effect of ZnO_x_ on the hydrocarbon FE is only present in the first 2 h of electrolysis when ZnO has not been fully reduced to Zn. Hence, we postulate that the Ni/ZnO_x_ interface leads to partially oxidized Ni, which promotes hydrocarbon formation on Ni. After this time, the NiO electrode exhibits a higher FE for hydrocarbons than the NiZnO ones, attributed to the formation of a NiZn alloy phase that promotes the competing hydrogen evolution reaction. Besides these selectivity trends, the nickel‐containing samples show a high structural stability and a rather surprisingly increasing selectivity toward hydrocarbon over time, whereas zinc is prone to leaching. While suppressing the competing hydrogen evolution reaction remains an important challenge, our work demonstrates that Ni‐based catalysts are an interesting option for stable CO_2_ electroreduction to high‐value hydrocarbon products.

## Supporting information

Supplementary Material
