# Supplementary material for: Carbon‐Supported NiZnO Nanoparticles for Electrochemical Reduction of CO2 to Hydrocarbons
Source: Chemphyschem. 2026 Jun 5;27(11):e202500486. doi: 10.1002/cphc.202500486 (PMC13241295; doi:10.1002/cphc.202500486)
Supplement: Supplementary file 1 — Supplementary Material [file CPHC-27-e202500486-s001.pdf]

Supporting Information

Carbon-supported NiZnO nanoparticles for electrochemical reduction of CO_2 to hydrocarbons

Matt L. J. Peerlings[1], Floris S. J. van Seters[1], Nienke L. Visser[1], Alexander P. Flick[2], Erik Betz-Güttner[1], Ramon van Maanen[1], Emiel J. M. Hensen,[2] Petra E. de Jongh[1], Peter Ngene[1]*

1. Materials Chemistry and Catalysis, Debye Institute for Nanomaterials Science, Utrecht University, 3584 CG Utrecht, The Netherlands
2. Laboratory of Inorganic Materials and Catalysis, Department of Chemical Engineering and Chemistry, Eindhoven University of Technology, P.O. Box 513, 5600 MB Eindhoven, The Netherlands

* Email: P.Ngene@uu.nl

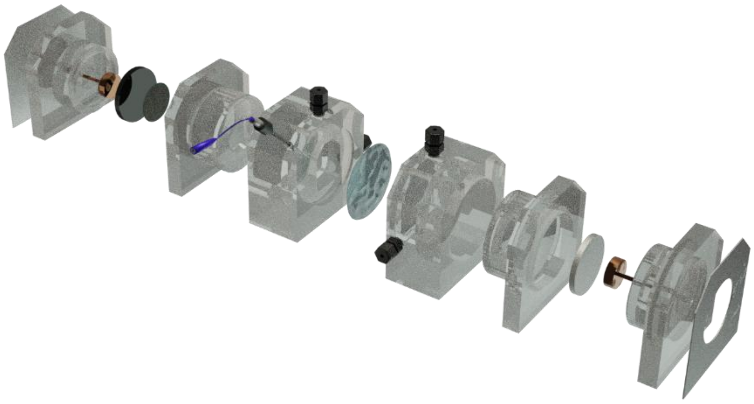

Figure S1. Custom-made H-type electrochemical cell used for catalytic tests. From left to right: glassy carbon disc on which the NiZnO/GNP-500 catalyst is spray coated, cathode compartment containing an Ag/AgCl reference electrode, Fumasep FAA-3-PK-130 anion exchange membrane, anode compartment and commercial IrO_2-based counter electrode pressed against a glassy carbon current collector.

Table S1. Overview of the number-averaged particle size of all NiZnO catalysts as determined using HAADF-STEM.

Catalyst	$d_n \pm \sigma_n$ (nm)
	**HAADF-STEM**
NiO	4.6 ± 1.0
$Ni_{20}Zn_1O$	5.0 ± 1.3
$Ni_{10}Zn_1O$	4.9 ± 1.1
Ni_5Zn_1O	5.2 ± 1.4
ZnO	n.a.

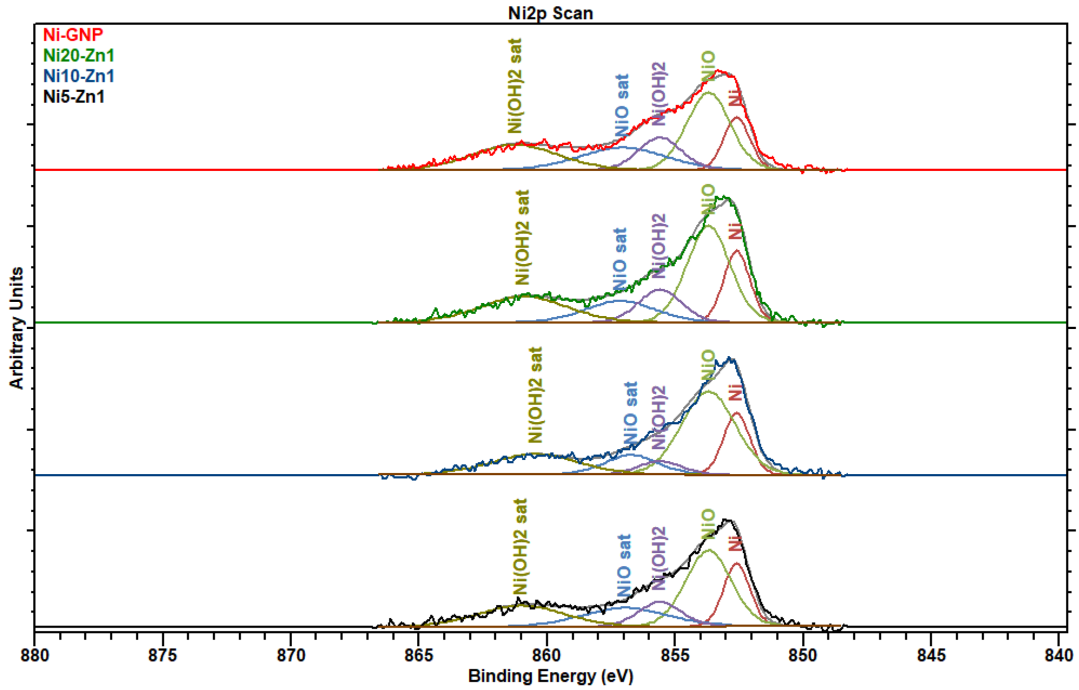

Figure S2. XPS spectra in the Ni $2p_{3/2}$ region, together with corresponding peak fittings for all nickel-containing as-synthesized catalyst powders.

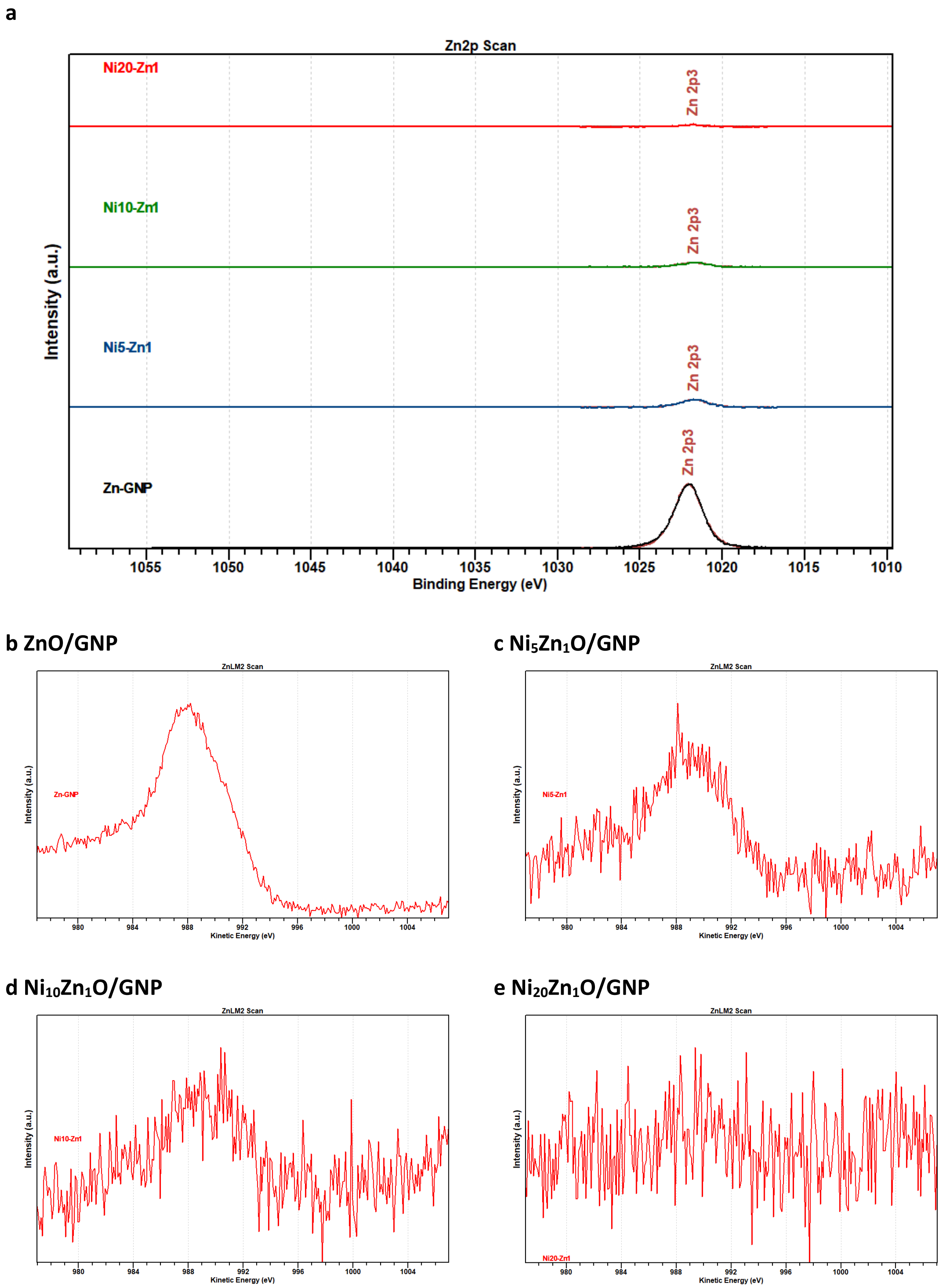

Figure S3a. XPS spectra in the Zn $2p_{3/2}$ region for all ZnO-containing catalysts. Zn LM2 spectra for **b.** ZnO/GNP, **c.** Ni_5Zn_1O/GNP, **d.** $Ni_{10}Zn_1O$/GNP and **e.** $Ni_{20}Zn_1O$/GNP catalysts, confirming the presence of ZnO for all catalysts.

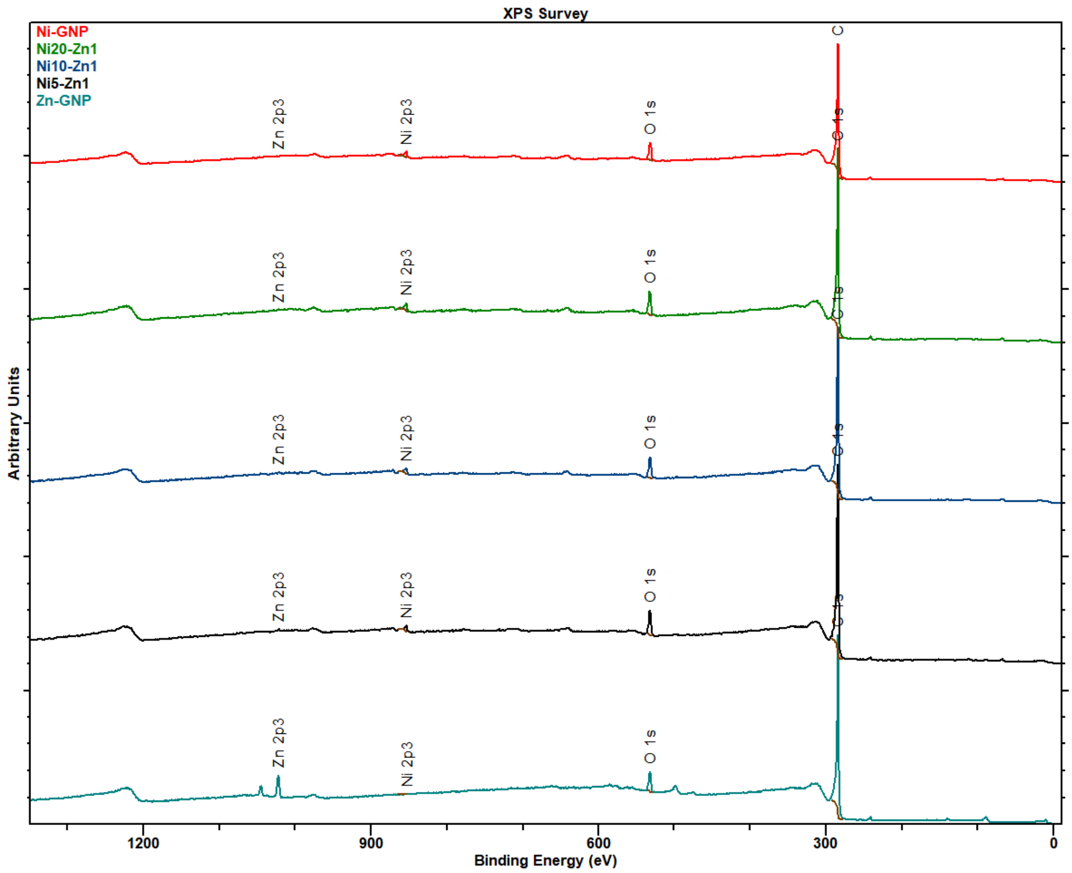

Figure S4. XPS survey spectra, confirming the presence of C, O, Ni and Zn in the as-synthesized catalyst powders. No contamination was observed.

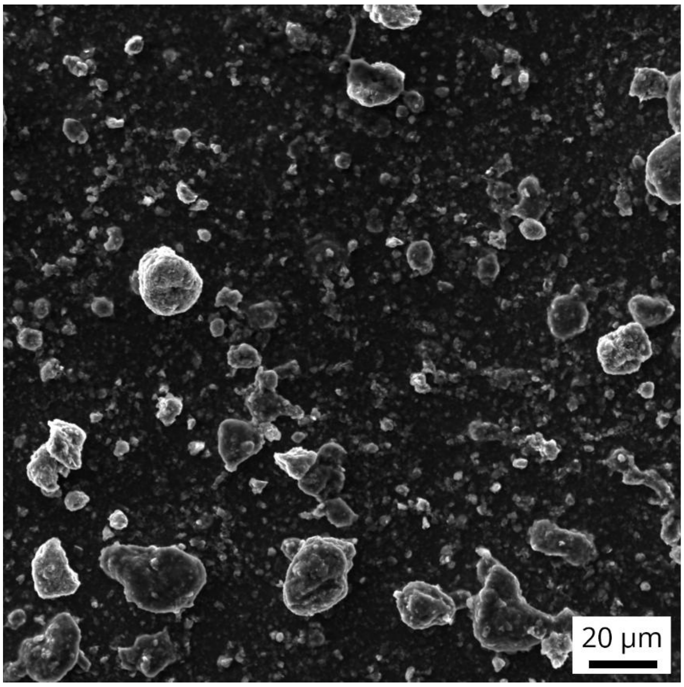

Figure S5. SEM image of the NiO/GNP electrode, consisting of the catalyst powder spray coated onto a glassy carbon disc.

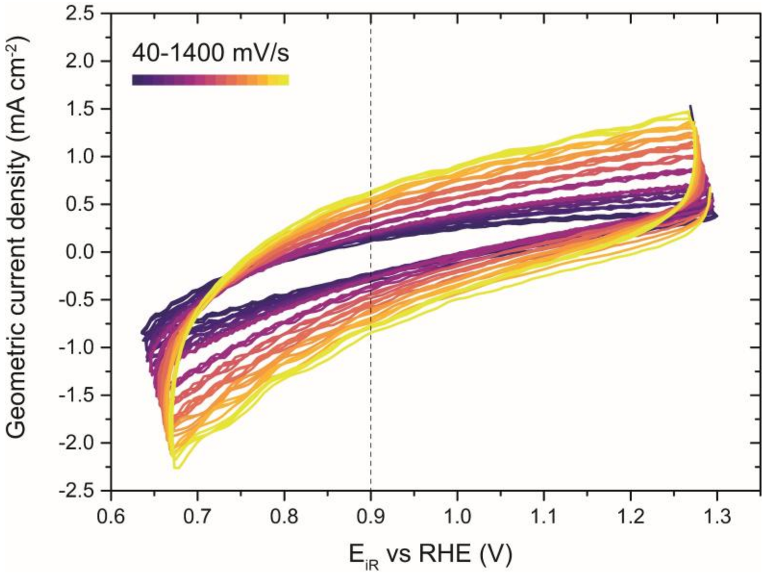

Figure S6. Representative double layer capacitance measurement of $Ni_{20}Zn_1O$/GNP electrode measured from +0.6 to +1.3 V at increasing scan rates of 40 (purple) to 1400 (yellow) mV s^{-1}.

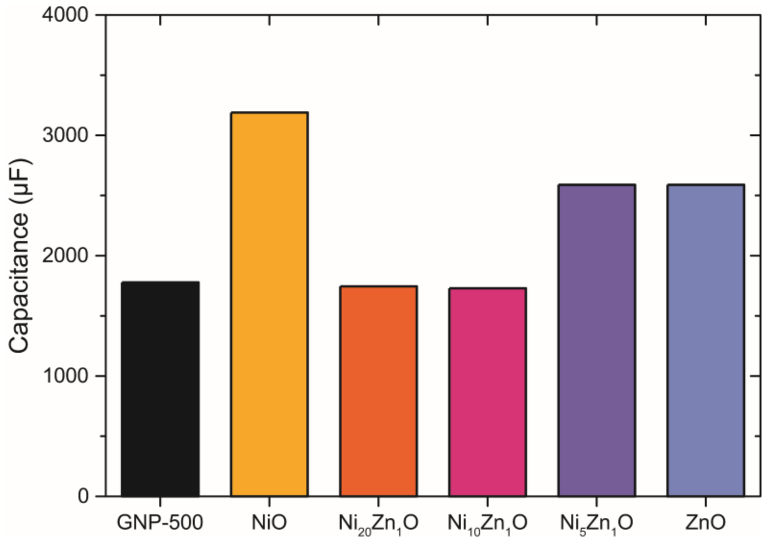

Figure S7. Overview of Double Layer Capacitance data obtained on the various electrodes and a reference electrode containing only the high surface area GNP-500 carbon support.

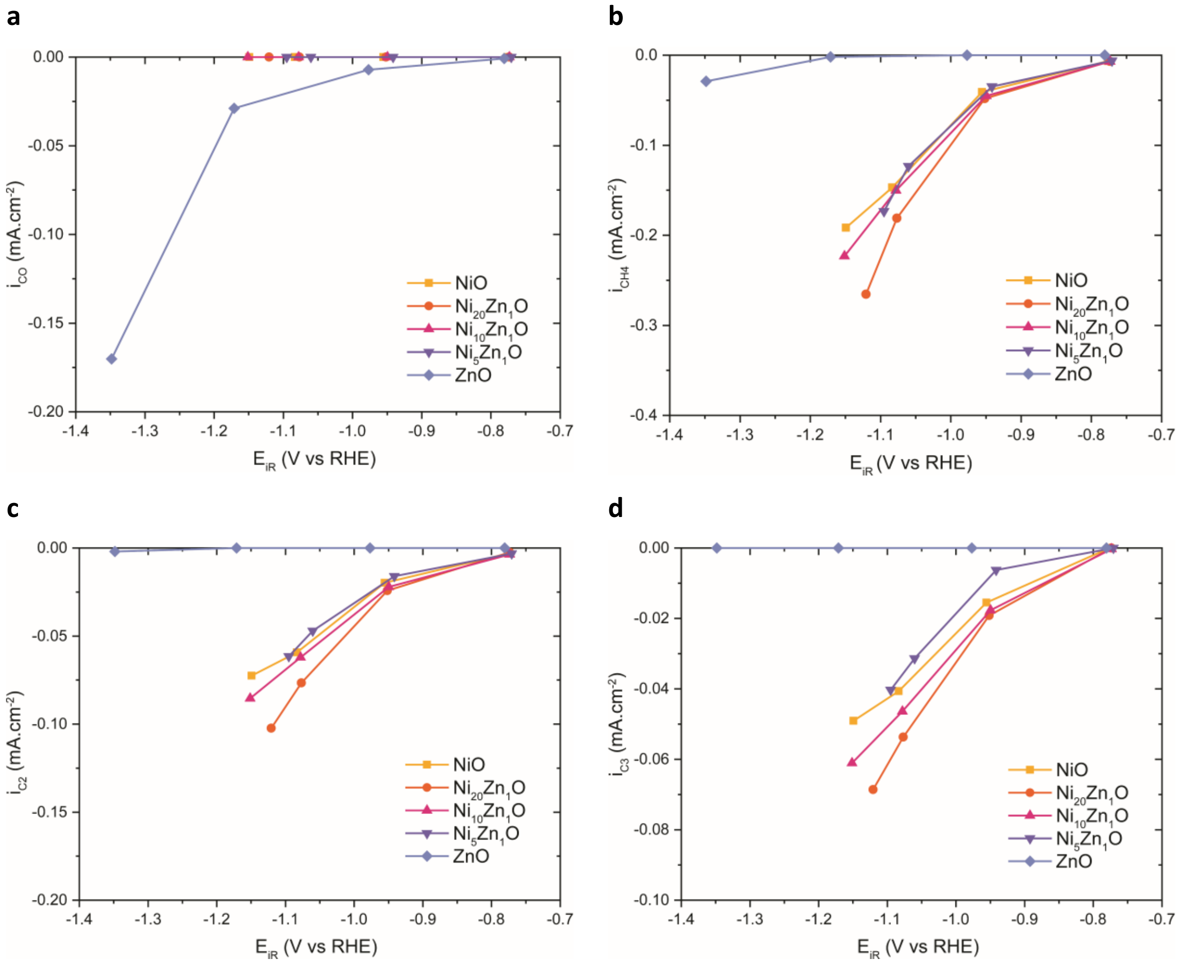

Figure S8. Partial current density to **a.** CO, **b.** CH_4, **c.** C_2 (ethylene and ethane) and **d.** C_3 (propylene and propane) hydrocarbons as function of potential.

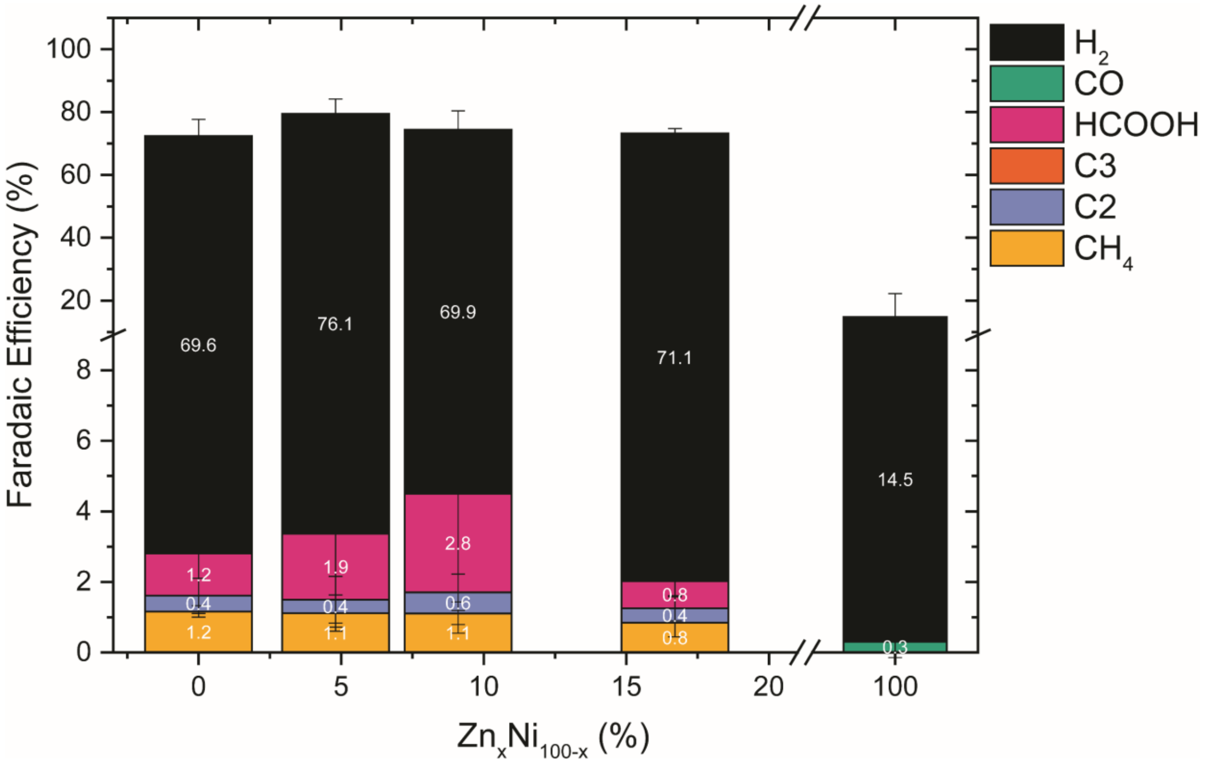

Figure S9. Faradaic Efficiency of the five NiZnO catalysts at -0.8 V vs RHE as function of atomic composition. C2 includes the ethylene and ethane, whereas C3 includes propylene and propane. The break on the y-axis is added to allow for better comparison of the CO_2RR product selectivity between electrodes due to the competing hydrogen evolution reaction (HER) being dominant.

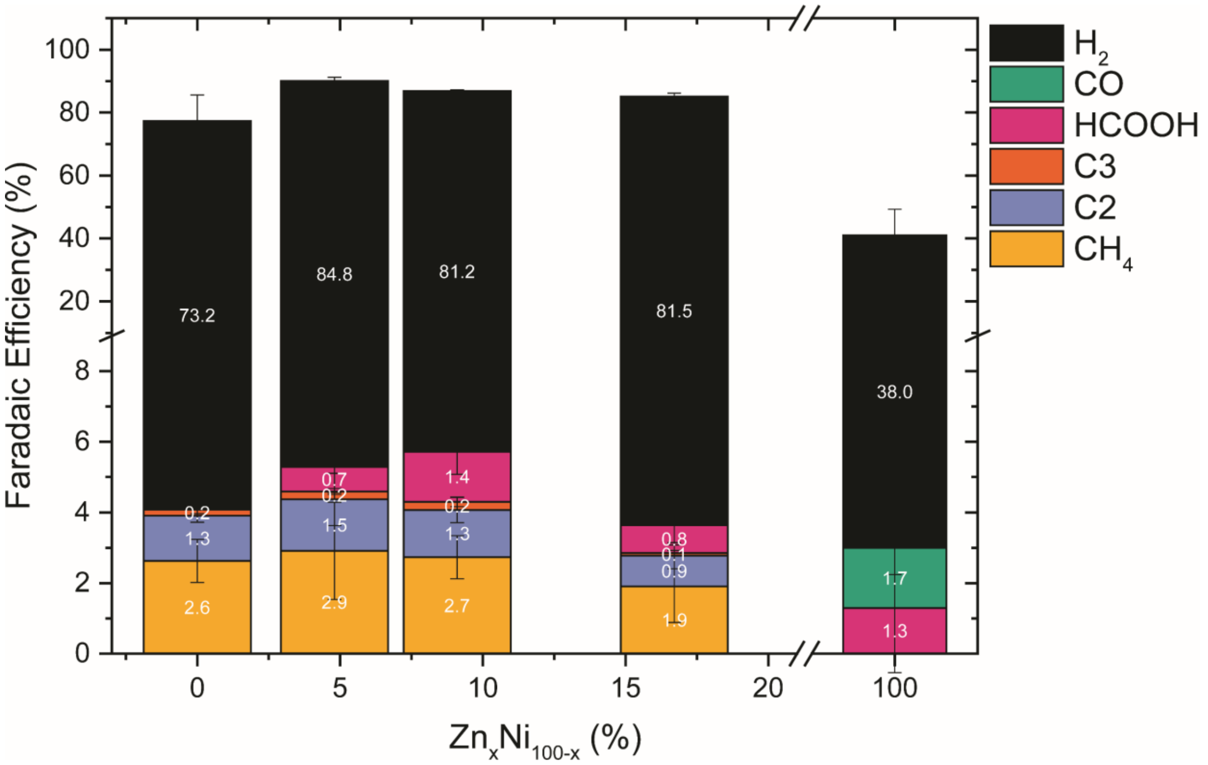

Figure S10. Faradaic Efficiency of the five NiZnO catalysts at -1.0 V vs RHE.

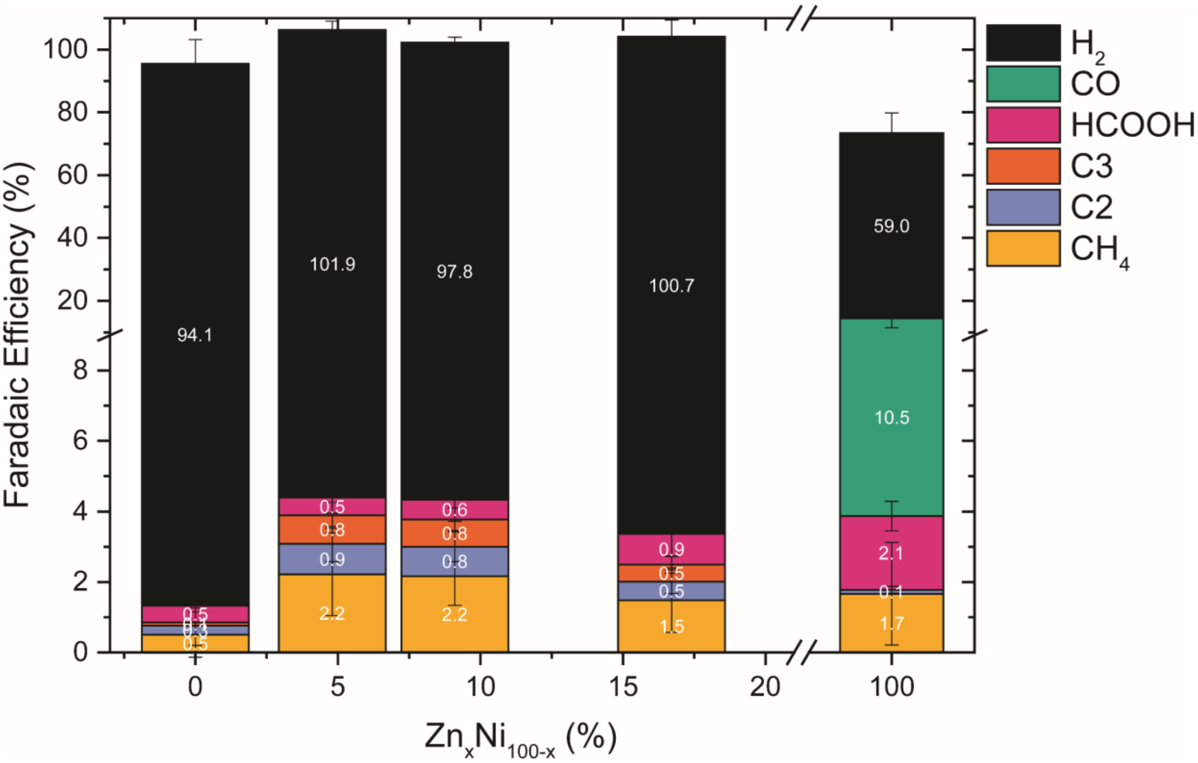

Figure S11. Faradaic Efficiency of the five NiZnO catalysts at -1.2 V vs RHE.

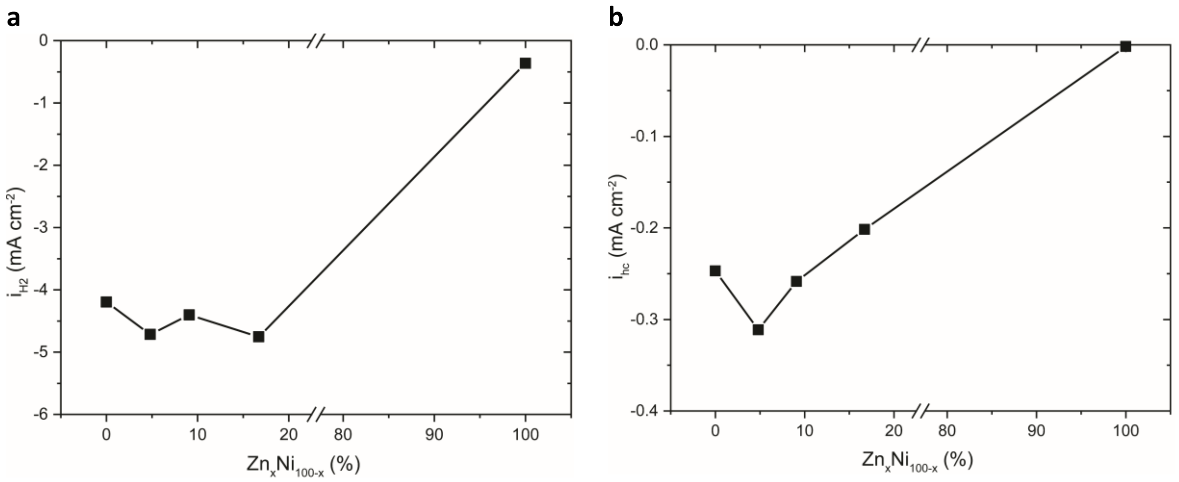

Figure S12. a. H_2 and **b.** hydrocarbon partial current densities as a function of atomic composition (expressed as Zn_xNi_{100-x}) at -1.1 V vs RHE.

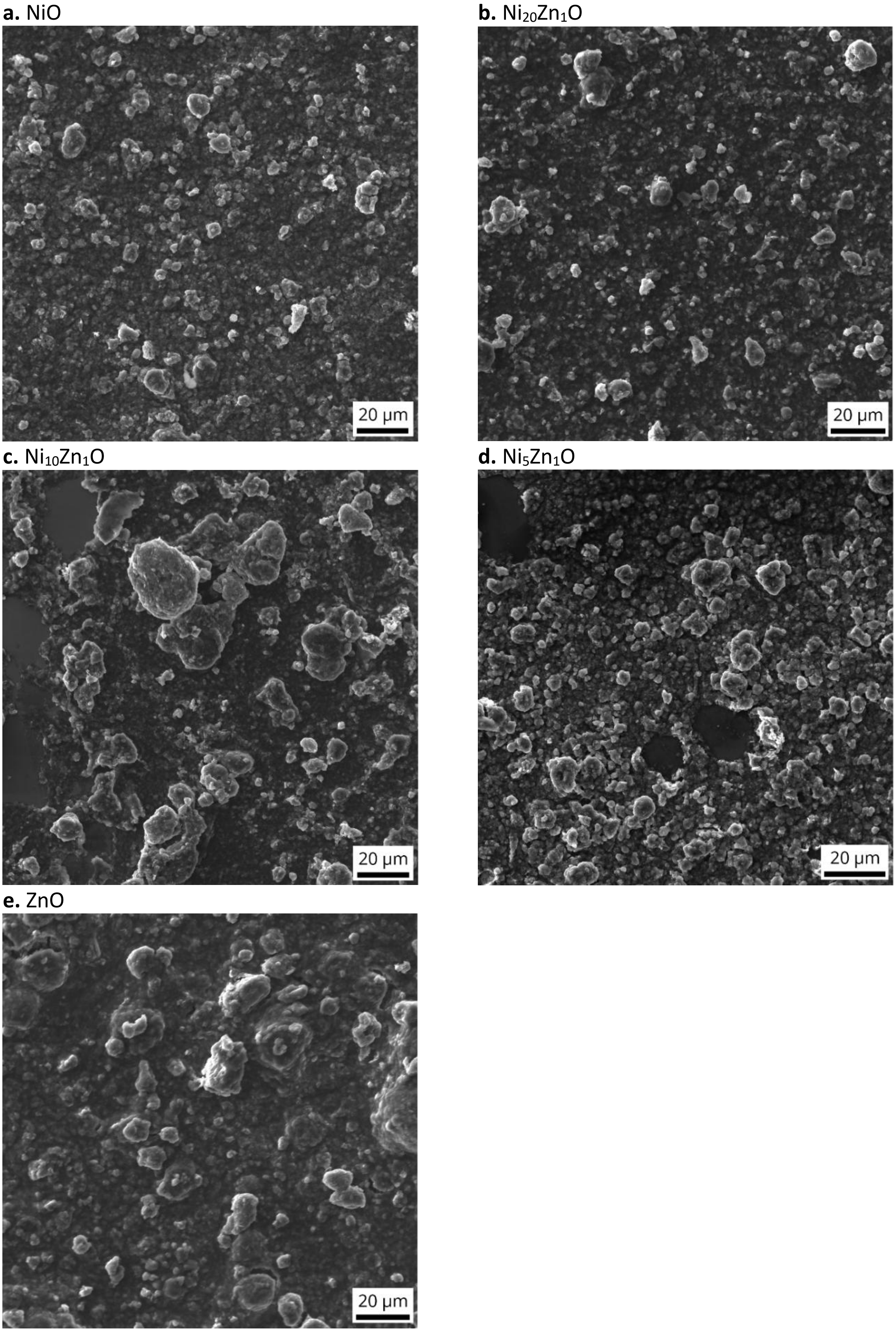

Figure S13. SEM images of **a.** NiO, **b.** $Ni_{20}Zn_1O$, **c.** $Ni_{10}Zn_1O$, **d.** Ni_5Zn_1O and **e.** ZnO electrodes after catalytic testing. The microstructures correspond to that of the GNP-500 carbon support. No (bi)carbonate deposits, nickel or zinc agglomerates are observed.

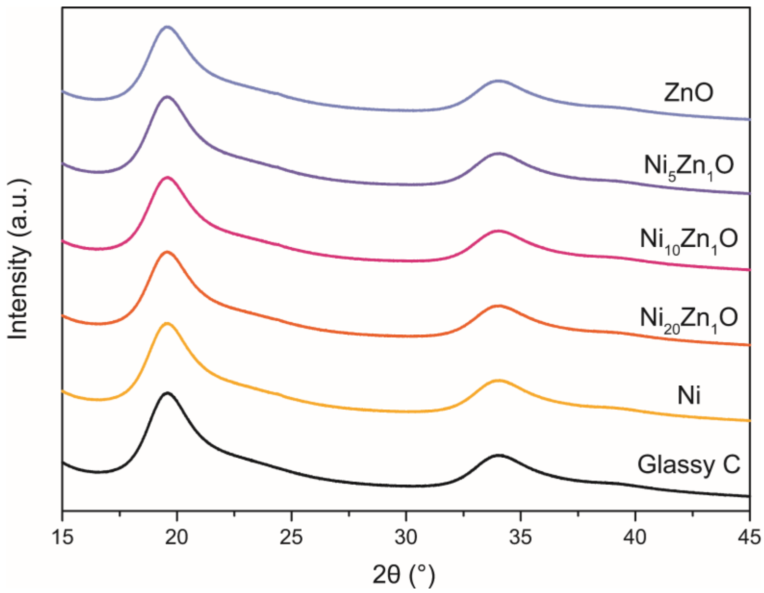

Figure S14. XRD patterns of the five NiZnO electrodes with different composition after catalytic testing. Note that the diffraction peaks have shifted because a Mo Kα X-Ray source was used instead of the Co Kα radiation that was used on the powders before catalytic testing. The diffractograms correspond fully to that of glassy carbon, which obscures all other signals due to its high crystallinity.

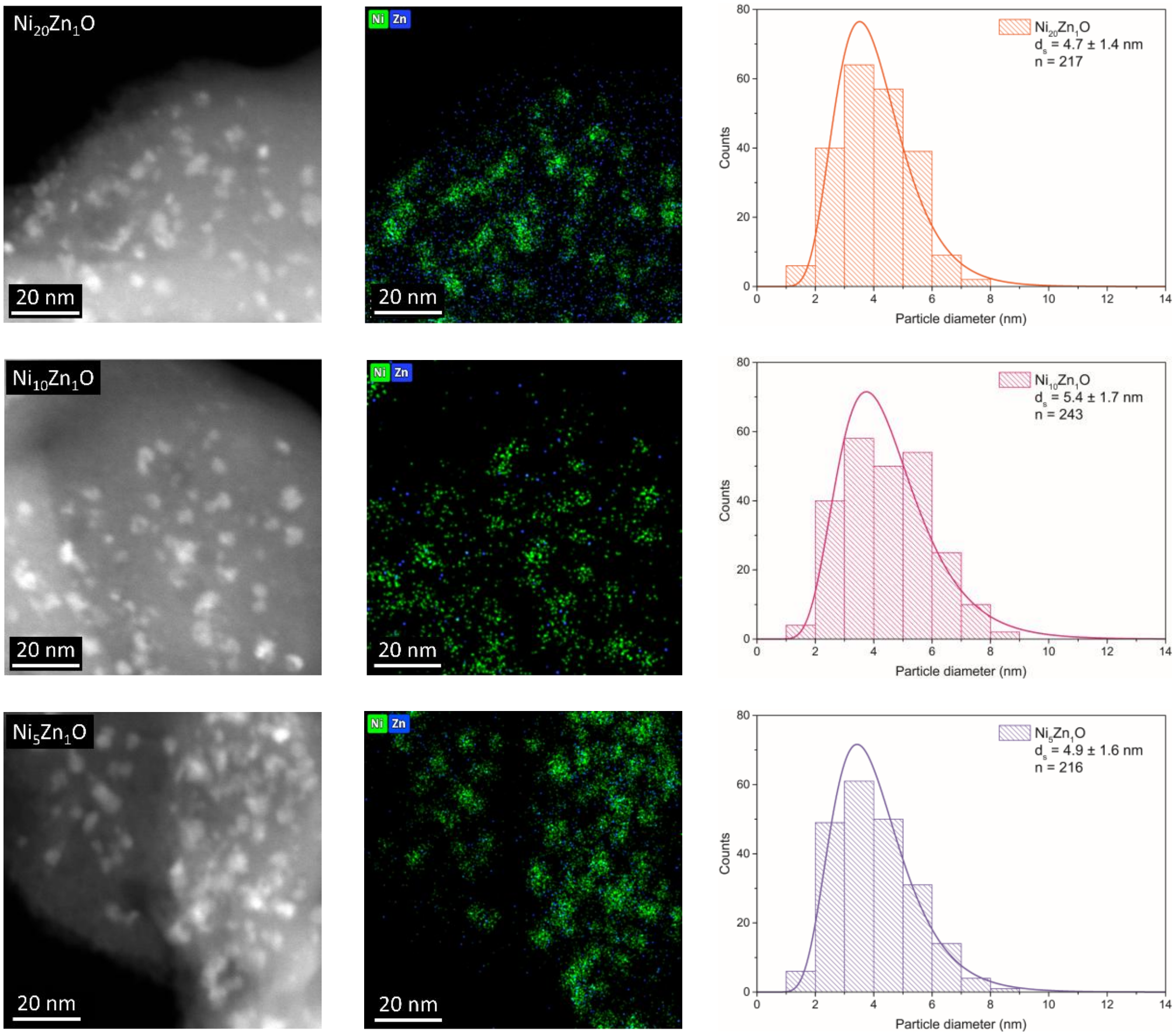

Figure S15. High resolution HAADF-STEM images (left) and corresponding EDX maps (middle), together with corresponding particle size distributions (right) of the bimetallic NiZnO samples after catalytic testing at five different potentials up to -1.2 V vs RHE for half an hour each.

Table S2. Overview of the Ni and Zn concentration as measured using ICP on the catholyte after catalytic testing at five different potentials.

**Catalyst**	**Concentration of Ni (ppm)**	**Concentration of Zn (ppm)**
NiO	0.19	-
$Ni_{20}Zn_1O$	0.02	0.04
$Ni_{10}Zn_1O$	0.05	0.06
Ni_5Zn_1O	0.15	0.03
ZnO	-	0.16

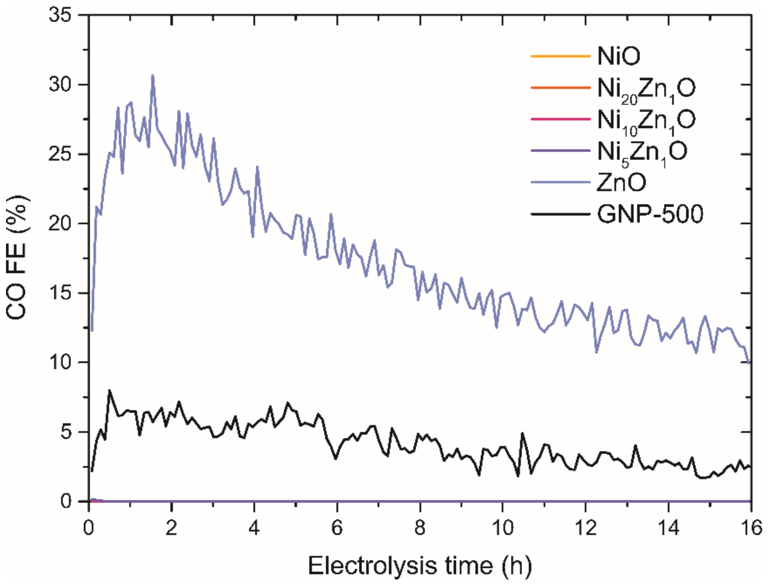

Figure S16. Faradaic Efficiency to CO as function of time for all tested electrodes.

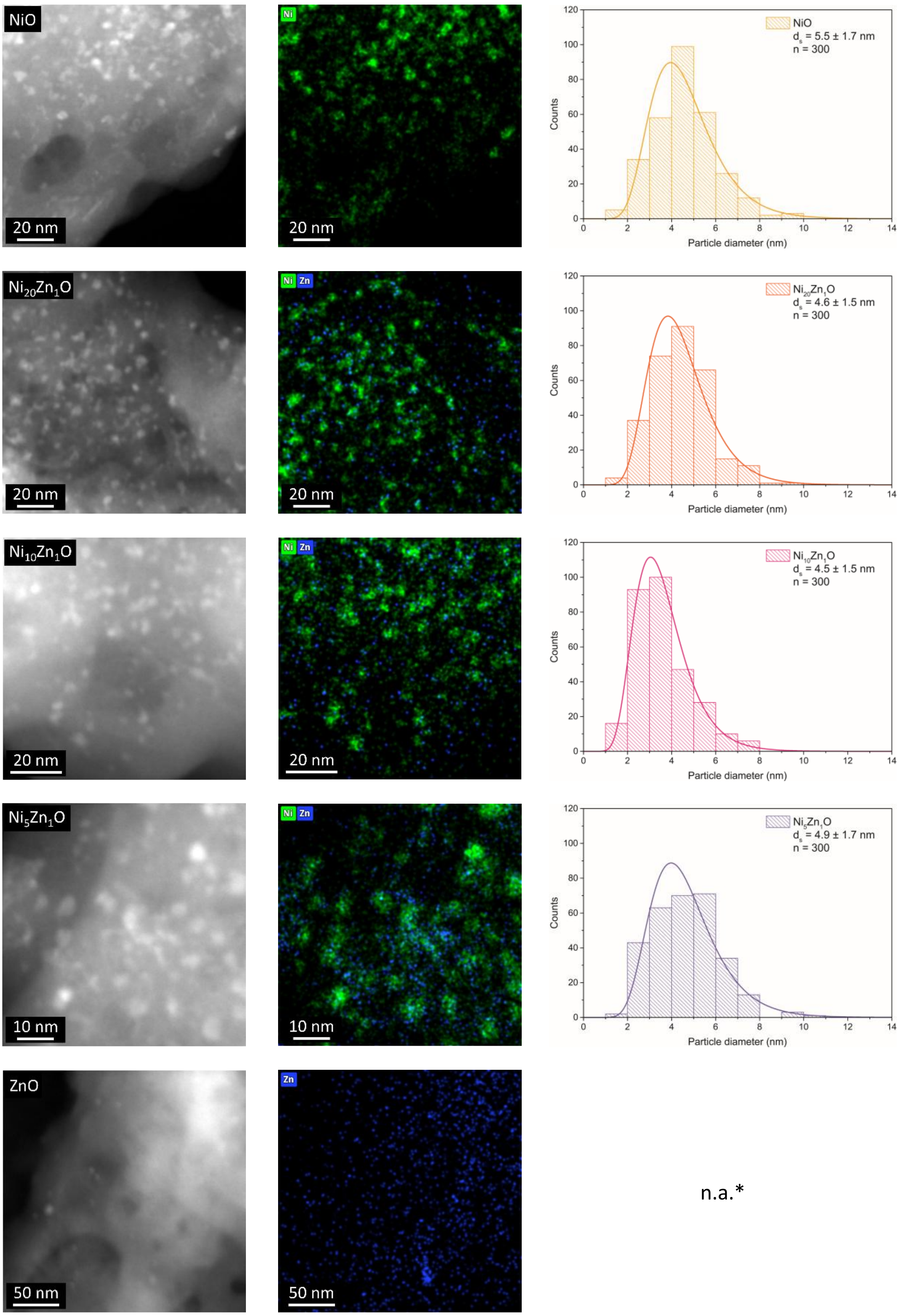

Figure S17. High resolution HAADF-STEM images (left) and corresponding EDX maps (middle), together with corresponding particle size distributions (right) of the NiZnO samples after catalytic

testing for 16 hours at -1.1 V vs RHE. *The absence of well-defined spherical particles for the ZnO sample made accurate particle size determination impossible.
